# Adverse childhood experiences and sources of childhood resilience: a retrospective study of their combined relationships with child health and educational attendance

**DOI:** 10.1186/s12889-018-5699-8

**Published:** 2018-06-26

**Authors:** Mark A. Bellis, Karen Hughes, Kat Ford, Katie A. Hardcastle, Catherine A. Sharp, Sara Wood, Lucia Homolova, Alisha Davies

**Affiliations:** 1 College of Health and Behavioural Sciences, Bangor University, Bangor, LL57 2UW UK; 2 Policy, Research and International Development Directorate, Public Health Wales, Clwydian House, Wrexham, LL13 7YP UK; 3 Policy, Research and International Development Directorate, Public Health Wales, Number 2 Capital Quarter, Tyndall Street, Cardiff, CF10 4BZ UK

**Keywords:** Adverse childhood experiences, Resilience, School attendance, Digestive diseases, Asthma

## Abstract

**Background:**

Adverse childhood experiences (ACEs) including maltreatment and exposure to household stressors can impact the health of children. Community factors that provide support, friendship and opportunities for development may build children’s resilience and protect them against some harmful impacts of ACEs. We examine if a history of ACEs is associated with poor childhood health and school attendance and the extent to which such outcomes are counteracted by community resilience assets.

**Methods:**

A national (Wales) cross-sectional retrospective survey (*n* = 2452) using a stratified random probability sampling methodology and including a boost sample (*n* = 471) of Welsh speakers. Data collection used face-to-face interviews at participants’ places of residence. Outcome measures were self-reported poor childhood health, specific conditions (asthma, allergies, headaches, digestive disorders) and school absenteeism.

**Results:**

Prevalence of each common childhood condition, poor childhood health and school absenteeism increased with number of ACEs reported. Childhood community resilience assets (being treated fairly, supportive childhood friends, being given opportunities to use your abilities, access to a trusted adult and having someone to look up to) were independently linked to better outcomes. In those with ≥4 ACEs the presence of all significant resilience assets (vs none) reduced adjusted prevalence of poor childhood health from 59.8 to 21.3%.

**Conclusions:**

Better prevention of ACEs through the combined actions of public services may reduce levels of common childhood conditions, improve school attendance and help alleviate pressures on public services. Whilst the eradication of ACEs remains unlikely, actions to strengthen community resilience assets may partially offset their immediate harms.

**Electronic supplementary material:**

The online version of this article (10.1186/s12889-018-5699-8) contains supplementary material, which is available to authorized users.

## Background

An increasing body of literature describes adverse childhood experiences (ACEs) and their impact on ill health later in adult life [1–3]. ACEs include suffering childhood abuse or neglect as well as environmental stressors such as living in a household affected by substance use or domestic violence. Chronic toxic stress resulting from ACEs can impact on the neurological, immunological and hormonal development of children [4, 5]. Repercussions of such impacts include substantive increases in risk of adopting anti-social and health-harming behaviours, accelerated development of chronic disease and premature death [1, 2]. Consequently, individuals with ≥4 ACEs in childhood (compared to those with none) are, as adults, more than twice as likely to smoke, nearly six times as likely to be problem alcohol users and over twice as likely to develop conditions such as cancer and heart disease [3]. These life course perspectives on ACEs expose their long-term health and financial costs to individuals and public services [6, 7]. Further, research is increasingly identifying more immediate impacts of ACEs on a wide range of health and social outcomes during childhood [8–10].

ACEs can result in direct physical damage to a child (e.g. fractures). However, children who have suffered or are suffering ACEs also experience higher levels of asthma [11] gastrointestinal conditions [12], headaches [13] and other somatic complaints [10] as well as lower overall levels of good health [8, 14]. Typically, increasing numbers of ACEs has a positive relationship with increasing health problems [10]. Developmental evidence supports ACEs as causal factors in such problems [4, 5]; although children with health difficulties are also at increased risk of ACEs [15]. Further, ACEs are associated with poorer childhood mental health, attendance at school, educational attainment and anti-social and violent behaviours [8, 16–18]. Such factors can be part of a life course that connects childhood adversity with long-term adult ill health.

Most children exposed to ACEs do not develop poor health outcomes. A range of factors may moderate the impact of ACEs on life course health, providing resilience to developmental harms and consequently, better outcomes despite a history of multiple ACEs [19]. Although many definitions are available [20], resilience typically describes the ability to adapt successfully to disturbances that threaten development of a positive life course or the ability to resume one following periods of adversity. Sources of resilience can include, but are not limited to, cultural engagement, community support, opportunity to control one’s personal circumstances and access to a trusted adult throughout childhood who can provide sanctuary from the chronic stress of ACEs [19, 21–23]. The concept of developing resilience in children as a moderator of ACE harms is widely advocated [19, 24, 25]. A range of interventions aim to enhance resilience through supporting parents; strengthening links with other family members, peers and schools; developing team working, decision-making abilities and confidence; and enhancing academic, athletic and other individual strengths [19, 26, 27]. However, relatively little empirical data is available on the extent to which resilience assets can counteract the impact of ACEs in childhood or which assets are most effective.

Using a national study on ACEs and resilience in Wales we examine if a history of ACEs is associated with increased levels of common childhood health conditions. We measure the relationships between ACEs and a history of childhood asthma, allergies, headaches and digestive disorders; each of which has previously been linked with childhood adversity [10–13] and is also a relatively common childhood condition in the study population. We examine the broader measures of overall poor childhood health status and poor school attendance. We test whether seven community resilience assets show protective relationships with each child health condition, overall health status and school attendance. Finally, we discuss which resilience assets have the potential to counter harms associated with chronic toxic stress.

## Methods

### Data collection

A core sample size of 2000 was set based on previous ACE studies [6] to capture an adequate sample with higher levels of ACEs (≥4) [3]. A boost sample from areas with higher levels of Welsh speakers (> 40% vs national average 19% [28]) was also included (target *n* = 500). A random probability sampling method was used to recruit a sample of residents living in Wales representative for geography and deprivation. Sampling was stratified based on Health Board area and, within each Health Board, by deprivation quintile at the Lower Super Output Area (LSOA, geographic areas with a population mean of approximately 1600). LSOAs were categorised into deprivation quintiles based on their ranking in the Welsh Index of Multiple Deprivation [29]. Letters were sent to each randomly selected household, providing study information and the option to opt out. Households were visited by trained interviewers (March–June 2017) and household members presented with an information sheet outlining the purpose of the study and explaining its confidential, voluntary and anonymous nature, and offered another option to opt out. Face-to-face interviews were completed using computer assisted personal interviewing, with sensitive questions self-completed. Only one individual from each household was eligible to participate (chosen based on next birthday). Study inclusion criteria were Welsh resident, aged 18–69 years and cognitively able to participate (i.e. judged by interviewers as capable of understanding the questions). All materials were available in English and Welsh. A total of 7515 households were sent letters and 887 (11.8%) opted out at that stage. However, to complete our target sample, it was only necessary to contact 4042 households. Of these, 645 were ineligible (e.g. outside the age range) and so were removed from the sample leaving 3397 eligible households. A further 888 declined at the doorstep and three interviews could not be completed, leaving 2506 individuals completing the study and a completion rate (at doorstep) of 73.8% (2506 agreeing from 3397). However, if all households opting out at the letter stage are also included this falls to 58.5% (2506 from 3397 + 887). For analyses undertaken here sample size was 2452 due to 54 individuals not completing all questions required.

### Questionnaire

Questions from the Centers for Disease Control and Prevention short ACE tool [30] and the Short Child Maltreatment Questionnaire [31] were used to retrospectively measure respondents’ exposure to ACEs < 18 years of age. ACEs were grouped into eleven types (see Additional file 1: Table S1) and respondents categorised as reporting 0, 1, 2–3 or ≥ 4 ACEs for analysis. Consistent with multiple ACE studies globally [3], for the purposes of this study the ACE count is used as an independent variable in order to examine a cumulative measure of childhood adversity and provide comparability to other ACE studies. The ACE tool has been validated as a reliable tool for retrospective assessment of adverse childhood experiences [32, 33]. However, we could not identify a tool validated for use with adults to retrospectively measure childhood community resilience assets or one that had been used in national surveys. Therefore, we used questions consistent with established resilience measures (Child and Youth Resilience Measure [34]) with the addition of measuring access to a trusted adult in childhood; a factor previously related to resilience and ACEs [22]. The community resilience assets measured were: knowing where to get help, having opportunities to apply one’s skills, being treated fairly, enjoying community culture, having supportive friends, having people to look up to, and having a trusted adult available (see Additional file 1: Table S1 for questions and responses categorised as having each asset). Common childhood conditions measured were asthma, allergies, headaches, and constipation/diarrhoea (as a single digestive conditions variable). Responses were dichotomised into never/rarely and sometimes/often for analysis. Self-rated overall health was reported as either excellent, very good, good, fair or poor and dichotomised into poor childhood health ‘yes’ (fair or poor) or ‘no’ (excellent, very good or good). High secondary school absenteeism was categorised as ‘yes’ (those missing > 20 days per year) and ‘no’ (missing ≤20 day per year).

### Statistical analysis

Statistical analyses used SPSS v24. Analyses employed chi-squared for initial bivariate analyses and logistic regression to examine independent relationships between ACEs, resilience factors, and outcomes of interest. Interactive terms between ACEs and resilience factors were included in logistic regression models. Best fit models were identified using inclusion of independent variables where they significantly (*P* < 0.05) improved the fit of the model to observed data (for pre-final iterations of models see Additional file 1: Table S2). Adjusted means for having each childhood health conditions, poor childhood health and high secondary school absenteeism were calculated based on best fit logistic regression models using the estimated marginal means function. Differences between adjusted means were tested using pairwise contrast (Wald Chi-squared test) functions [35].

## Results

Across the sample 48.5% of individuals reported at least one ACE (18.9% 1 ACE, 16.2% 2–3 ACEs, 13.4% ≥4 ACEs). All outcomes showed strong increases with ACE count. Digestive conditions, poor childhood health and school absenteeism showed the greatest relative increases between 0 and ≥ 4 ACEs categories (Table 1). With the exception of digestive conditions, each common childhood condition, poor childhood health and school absenteeism decreased with age. Women reported higher levels of allergies, headaches and digestive conditions. Overall poor childhood health and school absenteeism showed strong increases with deprivation (Table 1). Only asthma and school absenteeism showed a significant relationship with ethnicity (higher in those identifying as white; Table 1). Membership of the Welsh speaking boost sample was not related to any outcome.

**Table 1:** Relationship between childhood health and well-being status measures, ACE count and demographics

			Percentage with each condition/outcome					
			Common childhood conditions^a^				Poor childhood health^b^ %	High school Absenteeism^c^ %
		n	Asthma %	Allergies %	Headaches %	Digestive %		
All		2452	13.3	19.0	23.3	12.5	17.2	12.0
ACE count	0	1262	9.4	15.4	17.0	8.3	10.9	5.5
	1	464	12.0	17.9	24.1	14.0	15.9	11.6
	2–3	398	18.1	20.6	28.6	13.1	22.6	15.6
	≥4	328	24.1	32.3	40.2	25.9	36.9	32.9
	*X* ^2^		58.515	49.695	87.569	75.100	133.594	190.809
	P		< 0.001	< 0.001	< 0.001	< 0.001	< 0.001	< 0.001
Age (years)	18–29	437	21.5	22.7	32.0	12.6	24.0	14.0
	30–39	452	18.1	22.3	25.0	11.3	18.4	14.2
	40–49	495	9.9	17.8	24.4	12.9	16.8	13.3
	50–59	505	11.5	18.8	19.8	14.1	13.7	13.5
	60–69	563	7.6	14.6	17.6	11.7	14.6	6.2
	*X* ^2^		56.814	14.786	33.362	2.128	21.967	23.292
	P		< 0.001	0.005	< 0.001	0.712	< 0.001	< 0.001
Sex	Male	1115	13.9	17.0	19.6	10.8	16.4	12.3
	Female	1337	12.8	20.6	26.5	14.0	17.9	11.7
	*X* ^2^		0.652	5.394	16.304	5.770	0.914	0.171
	P		0.420	0.020	< 0.001	0.016	0.339	0.680
Deprivation quintile								
(least deprived) 1		459	11.5	17.2	19.4	10.9	12.2	6.3
	2	513	13.3	20.5	23.2	13.1	15.0	9.4
	3	614	12.9	19.9	24.4	14.5	18.1	9.9
	4	470	14.5	19.6	25.1	11.3	20.9	17.0
(most deprived) 5		396	14.6	16.9	24.2	12.1	20.2	19.2
	*X* ^2^		2.504	3.192	5.418	4.154	17.013	50.560
	P		0.644	0.526	0.247	0.386	0.002	<.0001
Sample^d^	Core	1981	13.5	18.7	23.6	12.1	17.1	12.5
	Boost	471	12.3	20.2	22.1	14.4	17.8	10.0
	*X* ^2^		0.487	0.552	0.507	1.956	0.159	2.235
	P		0.485	0.458	0.476	0.162	0.690	0.135
Ethnicity	White	2362	13.6	18.9	23.2	12.4	17.4	12.3
	Other	90	5.6	21.1	26.7	15.6	13.3	4.4
	*X* ^2^		4.855	0.280	0.582	0.786	0.986	5.041
	P		0.028	0.597	0.445	0.375	0.321	0.025

^a^Positive response: sometimes or often suffered each condition; ^b^Self-rated overall health in childhood was categorised ‘poor’ for responses fair or poor; other options were excellent, very good or good; ^c^Secondary school absenteeism was rated high if > 20 days per year; ^d^Core sample was randomly stratified nationally, boost was randomised across high Welsh speaking areas (see methods)

Using logistic regression analysis (Table 2) to account for relationships with demographics, ACEs remained strongly related to all outcomes. For all outcomes increases were significant even between those with 0 and 1 ACE, except for asthma and allergies where differences were significant at the 2–3 ACEs level and above (vs 0 ACEs; Table 2). Females were more likely to report higher levels of digestive conditions and allergies and, in particular, headaches (Table 2). Only school absenteeism increased with deprivation. Asthma and school absenteeism were both lower in those self-categorising as not white. Likelihood of reporting asthma, headaches, poor childhood health and school absenteeism varied with age with lower levels reported typically in those aged 60–69 years.

**Table 2:** Logistic regression analysis presenting adjusted odds ratios for each childhood health and well-being outcome by ACE count and demographics

	Common childhood conditions^a^											
	Asthma		Allergies		Headaches		Digestive		Poor childhood health^b^		High school absenteeism^c^	
	AOR (95%CIs)	P	AOR (95%CIs)	P	AOR (95%CIs)	P	AOR (95%CIs)	P	AOR (95%CIs)	P	AOR (95%CIs)	P
ACE count												
0	Ref	***		***		***		***		***		***
1	1.25 (0.89–1.75)	0.205	1.17 (0.88–1.55)	0.290	1.48 (1.14–1.93)	**	1.78 (1.28–2.48)	***	1.51 (1.11–2.05)	**	2.15 (1.48–3.14)	***
2–3	1.91 (1.38–2.64)	***	1.35 (1.01–1.81)	*	1.83 (1.40–2.39)	***	1.64 (1.15–2.33)	**	2.33 (1.73–3.14)	***	2.79 (1.93–4.04)	***
≥ 4	2.76 (2.00–3.82)	***	2.47 (1.87–3.28)	***	3.03 (2.31–3.97)	***	3.80 (2.76–5.22)	***	4.68 (3.50–6.26)	***	7.24 (5.15–10.19)	***
Age (years)												
18–29	Ref	***		0.050		***		0.592		**		*
30–39	0.83 (0.59–1.17)	0.284	1.00 (0.73–1.38)	0.999	0.71 (0.53–0.96)	0.025	0.94 (0.62–1.42)	0.771	0.74 (0.53–1.04)	0.080	1.11 (0.74–1.65)	0.619
40–49	0.40 (0.28–0.59)	***	0.75 (0.54–1.04)	0.082	0.70 (0.52–0.93)	*	1.11 (0.74–1.64)	0.620	0.65 (0.47–0.91)	*	1.08 (0.72–1.60)	0.714
50–59	0.48 (0.34–0.69)	***	0.82 (0.60–1.13)	0.234	0.55 (0.40–0.74)	***	1.27 (0.86–1.88)	0.225	0.54 (0.38–0.76)	***	1.16 (0.78–1.73)	0.452
60–69	0.34 (0.23–0.51)	***	0.66 (0.47–0.92)	*	0.53 (0.39–0.71)	***	1.16 (0.78–1.72)	0.473	0.69 (0.49–0.96)	*	0.61 (0.38–0.96)	*
Sex^d^												
Male	1.15 (0.91–1.47)	0.224	0.81 (0.66–1.00)	*	0.69 (0.56–0.84)	***	0.77 (0.60–0.99)	*	0.95 (0.76–1.18)	0.617	1.16 (0.90–1.51)	0.258
Deprivation quintile												
1 (least deprived)	Ref	0.993		0.320		0.554		0.444		0.102		***
2	1.06 (0.71–1.57)	0.785	1.19 (0.85–1.66)	0.305	1.23 (0.89–1.69)	0.206	1.22 (0.82–1.82)	0.337	1.17 (0.80–1.72)	0.413	1.49 (0.91–2.45)	0.113
3	0.98 (0.66–1.44)	0.899	1.08 (0.78–1.50)	0.626	1.25 (0.92–1.70)	0.162	1.27 (0.86–1.87)	0.226	1.38 (0.96–1.99)	0.081	1.49 (0.93–2.40)	0.099
4	1.03 (0.69–1.53)	0.881	1.04 (0.74–1.47)	0.809	1.22 (0.89–1.69)	0.219	0.95 (0.63–1.45)	0.819	1.60 (1.11–2.32)	*	2.63 (1.66–4.18)	***
5 (most deprived)	0.99 (0.65–1.50)	0.963	0.81 (0.56–1.18)	0.271	1.06 (0.75–1.48)	0.744	0.97 (0.63–1.50)	0.900	1.45 (0.98–2.13)	0.062	2.85 (1.78–4.56)	***
Sample^e^												
Boost	0.94 (0.68–1.30)	0.723	1.06 (0.81–1.38)	0.677	0.90 (0.69–1.16)	0.403	1.13 (0.83–1.55)	0.431	1.08 (0.81–1.43)	0.603	0.89 (0.62–1.28)	0.533
Ethnicity^f^												
Other	0.32 (0.13–0.81)	*	1.12 (0.66–1.91)	0.673	1.11 (0.68–1.82)	0.672	1.46 (0.80–2.67)	0.216	0.70 (0.37–1.32)	0.272	0.32 (0.11–0.89)	*

Ref = reference category; AOR = Adjusted Odds Ratio; *P < 0.05, **P < 0.01, ***P < 0.001. ^a^Positive response: sometimes or often suffered each condition; ^b^Self-rated overall health in childhood was categorised ‘poor’ for responses fair or poor; other options were excellent, very good or good; ^c^Secondary school absenteeism was rated high if > 20 days per year; ^d^Reference group = female; ^e^Boost is additional randomised sample from high Welsh language areas (> 40% Welsh speaking), reference group = core sample; ^f^Reference group = White

Seven childhood community resilience assets were included in analysis. Nearly half (48.3%) of respondents reported all resilience assets with 9.7% reporting less than two assets. There were strong positive relationships between higher levels of access to each asset (rated quite a bit or a lot; Additional file 1: Table S1) and lower levels of reporting poor childhood health and high school absenteeism (Table 3). Having a role model, supportive friends, being culturally engaged or given opportunities were all significantly related to lower levels of all common childhood conditions. However, asthma was not significantly related to knowing where to get community help, having access to a trusted adult or feeling you were treated fairly. Digestive problems were also not significantly related to knowing where to get community help (Table 3). Individuals reporting lower childhood community resilience assets were also much more likely to report higher ACE counts (Table 3).

**Table 3:** Relationships between childhood community resilience assets, child health and well-being measures and ACE count

		Percentage with each condition/outcome																
		Common childhood conditions												ACE count				
Childhood resilience assets		Asthma		Allergies		Headache		Digestive		Poor childhood health^a^		High school absenteeism^b^		0	1	2–3	≥4	
		%	*X* ^*2*^ *P*	%	*X* ^*2*^ *P*	%	*X* ^*2*^ *P*	%	*X* ^*2*^ *P*	%	*X* ^*2*^ *P*	%	*X* ^*2*^ *P*	%	%	%	%	*X* ^*2*^ *P*
Community help	No	14.4	1.325	22.4	9.573	26.3	6.230	14.1	3.103	23.7	37.396	16.6	26.303	38.6	16.5	22.5	22.4	152.624
	Yes	12.7	0.250	17.2	0.002	21.8	0.013	11.7	0.078	13.8	< 0.001	9.6	< 0.001	58.2	20.2	13.0	8.7	< 0.001
Adult available	No	15.4	2.595	22.9	6.925	29.4	14.673	18.5	22.711	29.1	69.472	20.5	48.036	30.7	15.7	20.7	32.9	271.993
	Yes	12.7	0.107	17.8	0.009	21.6	< 0.001	10.8	< 0.001	13.8	< 0.001	9.6	< 0.001	57.4	19.8	15.0	7.8	< 0.001
Given opportunities	No	18.9	17.258	23.6	8.889	29.5	13.546	17.5	14.456	31.8	96.315	24.8	99.173	29.3	15.9	22.2	32.6	256.338
	Yes	11.8	< 0.001	17.8	0.003	21.7	< 0.001	11.2	< 0.001	13.4	< 0.001	8.6	< 0.001	57.3	19.7	14.7	8.3	< 0.001
Treated fairly	No	17.0	4.820	30.3	33.557	35.2	31.630	19.6	18.479	36.6	106.645	29.1	112.219	21.9	13.0	23.3	41.8	332.704
	Yes	12.7	0.280	17.1	< 0.001	21.4	< 0.001	11.4	< 0.001	14.0	< 0.001	9.2	< 0.001	56.3	19.9	15.1	8.7	< 0.001
Culturally engaged	No	18.1	12.628	24.7	13.322	30.8	19.834	16.7	10.092	31.0	84.623	21.5	53.904	30.0	16.7	21.3	32.0	232.332
	Yes	12.1	< 0.001	17.5	< 0.001	21.4	< 0.001	11.4	< 0.001	13.6	< 0.001	9.5	< 0.001	56.0	19.5	14.9	8.6	< 0.001
Supportive friends	No	18.2	7.596	25.7	10.844	34.8	26.966	19.4	16.001	38.6	117.287	26.6	74.639	28.8	11.3	23.8	36.1	201.740
	Yes	12.6	0.006	18.0	< 0.001	21.6	< 0.001	11.5	< 0.001	14.0	< 0.001	9.8	< 0.001	54.9	20.1	15.1	10.0	< 0.001
Role model	No	19.7	12.791	27.6	17.618	34.0	22.878	20.3	20.062	38.7	117.471	28.6	94.168	18.7	13.7	22.9	44.8	356.495
	Yes	12.4	< 0.001	17.7	< 0.001	21.8	< 0.001	11.4	< 0.001	14.0	< 0.001	9.5	< 0.001	56.3	19.7	15.3	8.8	< 0.001

For full description of each resilience factor see Additional file 1: Table S1. ^a^Self-rated overall health in childhood was rated ‘poor’ for responses fair or poor, other options were excellent, very good or good; ^b^Secondary school absenteeism was rated high if > 20 days per year

Logistic regression analysis was used to examine the independent relationships between childhood outcomes and both resilience assets and ACE counts; interactions between ACE count and each resilience asset were also included in the model to identify changes in relationships between outcomes and resilience at different ACE counts (Table 4). Increasing ACE count remained strongly and positively related to all outcomes; the strongest relationships being with digestive conditions and school absenteeism (Table 4). The specific resilience assets reaching significance varied with outcome (Table 4). Thus, no resilience assets were significantly related to asthma. Being treated fairly, having supportive friends and access to a trusted adult were negatively related to allergies, headaches and digestive conditions respectively. Poor childhood health was also reduced by having supportive friends as well as being given opportunities and having a role model. School absenteeism was negatively related to being given opportunities as well as being treated fairly in the community (Table 4). However, for each resilience asset negatively associated with poor health and well-being outcomes, interactive terms with ACEs were not significant and therefore the impact of such assets did not differ with ACE count.

**Table 4:** Logistic regression analysis presenting adjusted odds ratios for each childhood health and well-being outcome by ACE count, resilience assets and demographics

		Common childhood conditions								Poor childhood health^a^		High school absenteeism^b^	
		Asthma		Allergies		Headaches		Digestive					
		AOR (95%CIs)	P	AOR(95%CIs)	P	AOR(95%CIs)	P	AOR(95%CIs)	P	AOR(95%CIs)	P	AOR(95%CIs)	P
ACE Count	0	Ref	< 0.001		< 0.001		< 0.001		< 0.001		< 0.001		< 0.001
	1	1.25 (0.89–1.75)	0.205	1.14 (0.86–1.52)	0.360	1.48 (1.14–1.92)	0.003	1.75 (1.26–2.44)	< 0.001	1.41 (1.03–1.92)	0.031	2.04 (1.40–2.99)	< 0.001
	2–3	1.91 (1.38–2.64)	< 0.001	1.26 (0.94–1.69)	0.130	1.75 (1.33–2.28)	< 0.001	1.56 (1.09–2.23)	0.015	1.79 (1.31–2.44)	< 0.001	2.32 (1.59–3.39)	< 0.001
	≥4	2.76 (2.00–3.82)	< 0.001	2.05 (1.51–2.77)	< 0.001	2.71 (2.05–3.59)	< 0.001	3.32 (2.36–4.67)	< 0.001	2.59 (1.86–3.61)	< 0.001	4.74 (3.26–6.89)	< 0.001
Childhood resilience assets	Community help		0.498		0.660		0.633		0.503		0.719		0.411
	Adult available		0.760		0.838		0.572	0.73 (0.55–0.97)	0.030		0.182		0.595
	Given Opportunities		0.051		0.583		0.992		0.571	0.64 (0.49–0.85)	0.002	0.57 (0.42–0.79)	< 0.001
	Treated fairly		0.579	0.61 (0.46–0.81)	< 0.001		0.188		0.436		0.276	0.60 (0.42–0.85)	0.004
	Culturally engaged		0.302		0.892		0.370		0.866		0.247		0.428
	Supportive friends		0.434		0.916	0.67 (0.51–0.88)	0.004		0.263	0.49 (0.36–0.67)	< 0.001		0.123
	Role model		0.315		0.493		0.411		0.478	0.58 (0.42–0.79)	< 0.001		0.114
ACE count* Childhood resilience assets	Community help		0.241		0.886		0.306		0.322		0.274		0.530
	Adult available		0.231		0.215		0.530		0.226		0.396		0.147
	Given Opportunities		0.369		0.847		0.600		0.240		0.904		0.339
	Treated fairly		0.526		0.926		0.155		0.291		0.480		0.479
	Culturally engaged		0.686		0.811		0.317		0.833		0.967		0.213
	Supportive friends		0.755		0.834		0.362		0.774		0.077		0.144
	Role model		0.717		0.835		0.754		0.195		0.574		0.579
Age (years)	18–29	Ref	< 0.001		0.046		< 0.001		0.641		0.002		0.042
	30–39	0.83 (0.59–1.17)	0.284	0.99 (0.72–1.36)	0.931	0.71 (0.53–0.96)	0.025		0.265	0.73 (0.52–1.02)	0.068	1.08 (0.72–1.63)	0.697
	40–49	0.40 (0.28–0.59)	< 0.001	0.73 (0.52–1.01)	0.055	0.68 (0.50–0.91)	0.010		0.904	0.57 (0.40–0.80)	< 0.001	0.99 (0.66–1.48)	0.942
	50–59	0.48 (0.34–0.69)	< 0.001	0.82 (0.60–1.14)	0.239	0.55 (0.40–0.74)	< 0.001		0.215	0.51 (0.36–0.73)	0.000	1.15 (0.77–1.72)	0.484
	60–69	0.34 (0.23–0.51)	< 0.001	0.66 (0.47–0.92)	0.013	0.52 (0.39–0.71)	< 0.001		0.705	0.65 (0.46–0.91)	0.012	0.59 (0.37–0.93)	0.023
Sex^c^	Male		0.233	0.79 (0.64–0.98)	0.030	0.68 (0.56–0.83)	< 0.001	0.76 (0.59–0.98)	0.031		0.341		0.303
Deprivation quintile	1 (least deprived)	Ref	0.991		0.232		0.677		0.236		0.144		< 0.001
	2		0.701		0.163		0.677		0.395		0.189	1.44 (0.87–2.37)	0.153
	3		0.696		0.462		0.452		0.067		0.375	1.51 (0.93–2.43)	0.094
	4		0.834		0.981		0.590		0.210		0.106	2.55 (1.60–4.06)	< 0.001
	5 (most deprived)		0.895		0.038		0.479		0.316		0.664	2.76 (1.72–4.44)	< 0.001
Sample^d^	Boost		0.685		0.289		0.695		0.174		0.269		0.723
Ethnicity^e^	Other	0.32 (0.13–0.80)	0.015		0.608		0.553		0.271		0.525		0.056

Ref = reference category. Logistic regression used a conditional model with only terms significantly contributing to the model’s fit to observed data (*P* < 0.05) retained in the final model. *P* values are shown for all terms entered into the model. AORs (Adjusted Odds Ratios) are only calculated for significant terms which remained in the model. Successive stages in model development for each outcome are shown in Additional file 1: Table S2. ^a^Self-rated overall health in childhood was categorised ‘poor’ for responses fair or poor; other options were excellent, very good or good; ^b^Secondary school absenteeism was rated high if > 20 days per year; ^c^Reference group = female; ^d^Boost is additional randomised sample from high Welsh language areas (> 40% Welsh speaking), reference group = core sample; ^e^Reference group = White

**Fig. 1:**
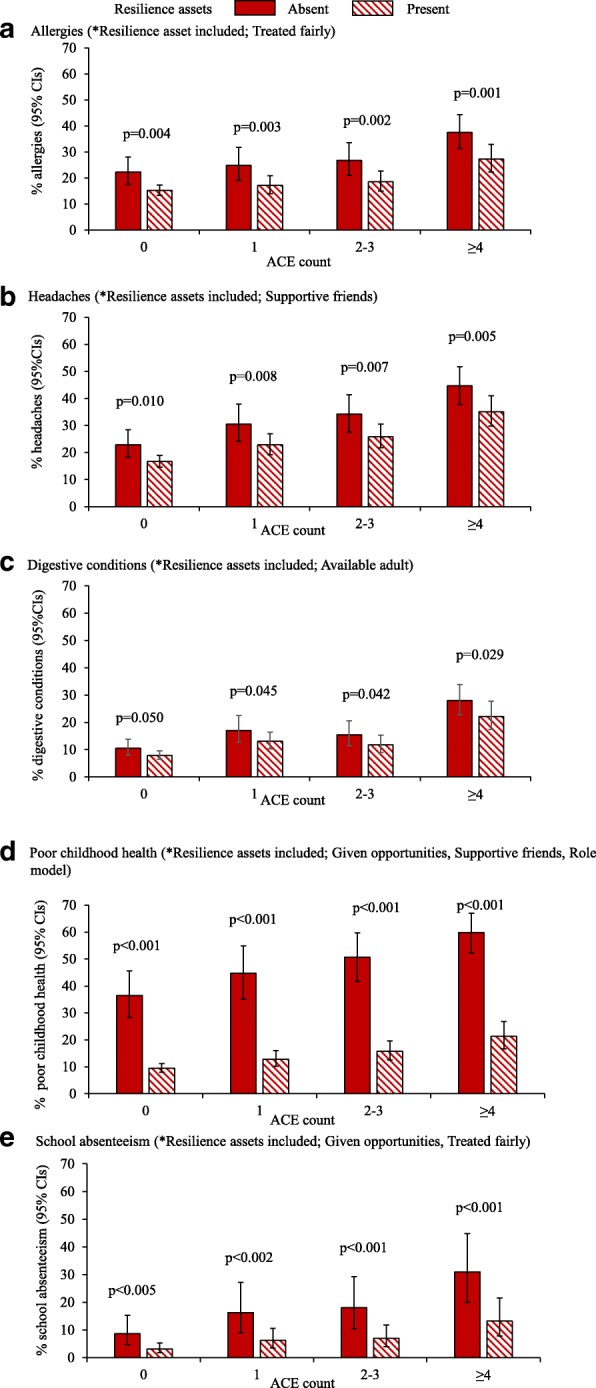
Adjusted mean^$^ percentage with each childhood health and well-being outcome by ACE count, stratified by childhood resilience assets* Footnote: ^$^Adjusted means are calculated using all significant terms in the logistic regression models for each outcome (see Table 4). *Only resilience assets significantly contributing to each model were included and these set to present or absent for each ACE category. Differences between columns (resilience assets absent or present) are tested using a pairwise contrast (Wald Chi-squared test, see Methods). No figure is included for asthma as there was no significant impact of any resilience assets (see Table 4)

Best fit logistic regression models (see methods) were used to estimate absolute differences in childhood health and well-being outcomes by ACE count; stratified by high and low resilience (i.e. all significant childhood resilience assets vs. no significant childhood resilience assets; Table 4). For all childhood health and well-being status measures (excluding asthma where no resilience assets were significant; Table 4), reporting high resilience assets was associated with better outcomes. For allergies and headaches differences in adjusted prevalence (between having and not having resilience assets) were significant (using pairwise contrasts) in all categories of ACE count (Fig. 1). Thus, levels of childhood allergies in those with 2–3 ACEs dropped from 26.8% (no resilience assets) to 18.6% when individuals felt treated fairly in their communities as a child. For digestive conditions the difference between prevalence with and without resilience assets did not quite reach significance in those with no ACEs but was significant at other ACE counts (Fig. 1). Poor childhood health and high school absenteeism also differed significantly depending on the presence of resilience assets in all ACE count categories (Fig. 1). Estimated percentage of individuals with poor childhood health, (in those with ≥4 ACEs) reduced from 59.8 to 21.3% when the assets being given opportunities, supportive friends and a role model were present. High school absenteeism, fell from 16.2 to 6.2% in those with one ACE when reporting no and all significant resilience assets respectively (i.e. given opportunities, treated fairly; Fig. 1).

## Discussion

We identified strong relationships between ACE counts and common childhood health conditions. For asthma, headaches and digestive conditions results show increases in prevalence with ACEs similar to those identified for childhood conditions in other studies; despite methodological differences in data collection [9–11]. While we found no ACE studies for allergies, previous work has described relationships between individual childhood stressors (e.g. physical abuse) and increased prevalence of allergic conditions [36]. Results here suggest ACEs are positively associated with childhood allergies; although the relative increase was not as great as for other conditions measured (Table 2). Common childhood conditions are a substantive component of pressures on primary care in the UK and other countries [37]. Consistent with an ACE aetiology, children presenting with such physical conditions often respond to interventions aimed at improving mental health rather than addressing their symptoms directly [38]. A larger mental health component in the development in children of conditions such as headaches and digestive conditions may in part underpin their stronger relationship with ACEs than asthma or allergies (Table 2) [3]. However, there has been little consideration of when or how ACE-related histories might be collected in general health care settings in order to better understand their contribution to common health conditions, reduce further childhood adversity or improve patient outcomes [39, 40].

Along with the physical conditions measured here, individuals with higher ACEs are also likely to suffer other (e.g. mental [17]) health and social problems. We used a broader measure of poor childhood health to capture wider impacts on health and also measured school absenteeism as a proxy for both health and social issues (e.g. ill health, school exclusion, truanting [41]). Proportions reporting poor childhood health increased more than three-fold from 0 to ≥4 ACE categories and approximately six-fold for high school absenteeism. Even in those with 2–3 ACEs (16.2% of people surveyed) levels of poor childhood health and high school absenteeism were more than doubled (vs. 0 ACEs, Table 1). Increases were maintained after correcting for potential demographic confounders (Table 2). Our results suggest schools might improve attendance by adopting approaches that tackle ACEs and their consequences. Some schools working with public health agencies have adopted ACE-informed approaches (i.e. staff understanding ACEs and adapting working practice to support children who have experienced them). This has increased attendance, reduced exclusions and improved educational attainment [42]. However, little has been done to adopt ACE-informed school models at scale.

With the eradication of ACEs a long-term goal, developing individual and community assets with the potential to counteract the negative outcomes associated with ACE exposure is an immediate consideration. Developing resilience has been widely considered as the principal component of such assets; despite little empirical data on its benefits. Results here support greater access to childhood resilience assets being strongly related to better health and well-being (Table 3) with greater childhood resilience assets having a positive impact on each health and well-being measure (Table 4). However, which resilience assets made significant contributions varied by outcome measure. Thus, no resilience assets measured here significantly affected levels of asthma whilst different assets were most strongly associated with each childhood health condition (e.g. supportive friends with lower prevalence of headaches; Table 4). Moreover, poor self-reported childhood health and high school absenteeism were both significantly related to multiple resilience assets. For poor childhood health status reporting all significant assets (i.e. given opportunities, having supportive friends and a role model) was associated with approximately a two thirds reduction in adjusted prevalence of poor childhood health across all categories of ACE count (Fig. 1). Similar benefits in school attendance across all ACE count categories were associated with being given opportunities but also being treated fairly in childhood communities. These findings suggest that those resilience assets measured here provide a similar level of benefit to individuals irrespective of their exposure to ACEs. Thus, having good role models, providing networking opportunities and settings for friendship building as well as ensuring a sense of fairness and equity in how children feel they are treated may not just be enhancements to childhood but could be essential to health and educational attendance. With all individuals appearing to benefit from accessing resilience assets and those with most ACEs presenting with the worst outcomes, well-being strategies should consider how resilience assets can be supported universally but to a greater extent where childhood adversity is concentrated.

However, this study faced a number of limitations. In particular, data were retrospective and self-reported and consequently may be affected by recall bias or unwillingness to report past experiences. An analysis of individual ACEs reported by age identified prevalence of some ACEs reducing with increasing age (e.g. verbal abuse, parental separation, drug and alcohol abuse) while others showed no clear age-related trend (e.g. domestic violence, sexual violence; Additional file 1: Table S3). Some ACEs are known to have increased in recent years (e.g. parental separation). However, separation is only a proxy measure for potentially chronic stress in family environments and the absence of separation may, in some cases, increase or extend periods of familial conflict. The extent to which trends in others (e.g. verbal abuse) are real or a function of recall bias cannot be established here. Studies comparing retrospective and prospectively collected measures of childhood adversity find both correlate with poor health outcomes in later life, with retrospective and prospective measures also showing moderate correlations with each other [43]. While retrospective measures inevitably risk incomplete or inaccurate recollection, prospective studies typically rely on reporting by caregivers or professionals and may also miss cases. Moreover, as prospective studies identify current child abuse or neglect they may intentionally or unintentionally alter its impact on current or later health outcomes. However, whilst we cannot measure or correct for variability introduced by using retrospective ACE, resilience and outcome measures this remains a limitation of the study. Further, our resilience measures showed similar beneficial relationships across those with and without ACEs. We cannot tell if such benefits related to resilience impacting unmeasured adversities in those with few or no ACEs or if resilience assets are beneficial regardless of ACE exposure. The study population was nationally selected using a random, stratified methodology. The generalisability of results to populations outside of Wales or those based in settings not usually accessed through door to door surveys (e.g. prisons, care settings) also cannot be established without further studies. Unlike ACEs, presently there is no validated tool for measuring cumulative resilience retrospectively. Consequently, we used a combination of questions from more commonly used tools including those previously examining associations with ACEs. Further, while this study identified resilience assets associated with improvement in each health and educational outcome (except asthma), which assets were significant varied with outcome (Table 4). This study could not elucidate why different resilience assets appear to protect against different harms; although this is an important area for further study. Finally, further studies with more detailed longitudinal data are also required to examine causality including examining the potential for reverse causality where those with better health subsequently adopt more resilience building opportunities.

## Conclusions

While ACEs can shape health across the life course, results here suggest their harmful impacts may manifest through increased common childhood health conditions as well as general poorer self-rated health. Such conditions are typically not life threatening but their long-term impact on physical and social development can be substantive. Thus, childhood ill health as well as anti-social behaviour impacts school attendance and consequently opportunities for educational attainment and better economic prospects throughout life. Integrated and trauma-informed (i.e. understanding, recognising and responding to the links between a history of trauma and current health and social problems) public services can provide support capable of preventing ACEs and potentially develop resilience assets that offset some of their harmful consequences [44]. Population surveys on ACEs can usefully inform national policy and the use of a combined ACE measure provides a framework for multi-agency engagement [45]. However, person level support for those at risk of, or exposed to, trauma require studies directly assessing the acceptability and effectiveness of interventions. Already parental support, early social skills development and pre-school enrichment interventions have all been associated with reduced risk of ACEs. For those who experience ACEs, trauma-informed public services are critical for understanding and addressing the underlying causes of health and behavioural problems seen in both educational and care settings. Currently, the eradication of ACEs remains beyond the scope of most communities. However, investment in assets that build resilience may counteract some of the harms disproportionately suffered by those with ACEs and results here suggest may also be of benefit to those with low or no ACEs. Many of the resilience assets examined in this study are features of thriving communities. Asset-based community development (ABCD) aims to enhance existing beneficial features within localities to enable residents to overcome the challenges they face [46]. An ABCD approach to resilience development would identify and invest in existing features that support cultural connectedness, friendship networks, community roles models and access to community support. The return on such investments could be substantive both in the short-term though improvements in child well-being and through the long-term gains from setting individuals on a healthier life course. While public policy and services consider how to effectively support such developments we should at the least ensure we do not disinvest from or dismantle community features that may be instinctively protecting some of the most vulnerable children.

## Additional file

Additional file 1: **Table S1.** Adverse childhood experience (ACE) and resilience questions with qualifying responses; **Table S2.** Pre-final model logistic regression analyses for each childhood health and well-being outcome by ACE count, resilience assets and demographics; **Table S3.** Prevalence of ACEs reported by age category of respondent. (DOCX 51 kb)

## Abbreviations

ABCD: Asset-based community development
ACEs: Adverse childhood experiences
LSOA: Lower Super Output Area
vs: Versus
