# Supplementary material for: Adverse childhood experiences and sources of childhood resilience: a retrospective study of their combined relationships with child health and educational attendance
Source: BMC Public Health. 2018 Jun 26;18:792. doi: 10.1186/s12889-018-5699-8 (PMC6020215; doi:10.1186/s12889-018-5699-8)
Supplement: Supplementary file 1 — Table S1. Adverse childhood experience (ACE) and resilience questions with qualifying responses; Table S2. Pre-final model logistic regression analyses for each childhood health and well-being outcome by ACE count, resilience assets and demographics; Table S3. Prevalence of ACEs reported by age category of respondent. (DOCX 51 kb) [file 12889_2018_5699_MOESM1_ESM.docx]

**Additional file 1**

**Table S1. Adverse childhood experience (ACE) and resilience questions with qualifying responses**

|  | | **Question** |  | **Qualifying response** | |
| --- | --- | --- | --- | --- | --- |
| **ACEs** | All ACE questions were preceded by the statement “While you were growing up, before the age of 18...” | | | |  |
| *Physical abuse* | | How often did a parent or adult in your home ever hit, beat, kick, or physically hurt you in any way? This does not include gentle smacking for punishment? |  | Once or more than once | |
| *Verbal abuse* | | How often did a parent or adult in your home ever swear at you, insult you, or put you down? |  | More than once | |
| *Sexual abuse* | | How often did anyone at least 5 years older than you (including adults) ever touch you sexually? |  | Once or more than once to any of the questions | |
|  | | How often did anyone at least 5 years older than you (including adults) try to make you touch them sexually? |  |  |  |
|  | | How often did anyone at least 5 years older than you (including adults) force you to have any type of sexual intercourse (oral, anal, or vaginal)? |  |  |  |
| *Physical neglect* | | Did your parent/caregiver for long periods of time not provide you with enough food or drink, clean clothes, or a clean and warm place to live when they could have? |  | Yes | |
| *Emotional neglect* | | Were there times when there was no adult living with you who made you feel loved? |  | More than once | |
| *Parental separation* | | Were your parents ever separated or divorced? |  | Yes | |
| *Domestic violence* | | How often did your parents or adults in your home ever slap, hit, kick, punch, or beat each other up? |  | Once or more than once | |
| *Mental illness* | | Did you live with anyone who was depressed, mentally ill, or suicidal? |  | Yes | |
| *Alcohol abuse* | | Did you live with anyone who was a problem drinker or alcoholic? |  | Yes | |
| *Drug abuse* | | Did you live with anyone who used illegal street drugs or who abused prescription medications? |  | Yes | |
| *Incarceration* | | Did you live with anyone who served time or was sentenced to serve time in a prison or young offenders' institution? |  | Yes | |
|  | |  |  |  | |
| **COMMUNITY RESILIENCE** | | When you were growing up, during the first 18 years of life, to what extent would the sentences below have described you?  *Response options: Not a lot, a little, somewhat, quite a bit, a lot. For adult available: never, sometimes or always* |  |  | |
| *Community help* | | I knew where to get help in my community |  | Quite a bit / a lot | |
| *Given opportunities* | | I had opportunities to apply my abilities in life (like skills, a job, caring for others) |  | Quite a bit / a lot | |
| *Treated fairly* | | I was treated fairly in my community |  | Quite a bit / a lot | |
| *Culturally engaged* | | I enjoyed my community’s cultures and traditions |  | Quite a bit / a lot | |
| *Supportive friends* | | My friends stood by me during difficult times |  | Quite a bit / a lot | |
| *Role model* | | I had people I looked up to |  | Quite a bit / a lot | |
| *Adult available* | | While you were growing up, before the age of 18, was there an adult in your life who you could trust and talk to about any personal problems?  *Response options: Never, sometimes, always* |  | Always | |

**Table S2. Pre-final model logistic regression analyses for each childhood health and well-being outcome by ACE count, resilience assets and demographics**

| **Common childhood conditions** | | | | | | | | | | | | | | | | | |  | |  |  | |  | | |  | |  | |
| --- | --- | --- | --- | --- | --- | --- | --- | --- | --- | --- | --- | --- | --- | --- | --- | --- | --- | --- | --- | --- | --- | --- | --- | --- | --- | --- | --- | --- | --- |
| **Asthma** | | | | **Allergies** | | | | **Headaches** | | | | | | **Digestive** | | | | **Poor childhood health** | | | | **High school absenteeism** | | | | | | | |
| **Model** | | **P** | **AOR** | **Model** | | **P** | **AOR** | **Model** | | **P** | | **AOR** | | **Model** | | **P** | **AOR** | **Model** | | **P** | **AOR** | | **Model** | | | **P** | | **AOR** | |
|  | Independent |  |  |  | Independent |  |  |  | Independent | |  | |  |  | Independent |  |  |  | Independent |  |  | |  | Independent |  | |  | |  |
| **1** | ACEs 0 | <0.001 |  | **1** | ACEs 0 | <0.001 |  | **1** | ACEs 0 | | <0.001 | |  | **1** | ACEs 0 | <0.001 |  | **1** | ACEs 0 | <0.001 |  | | **1** | ACEs 0 | <0.001 | |  | |  |
|  | 1 | 0.074 | 1.36 |  | 1 | 0.207 | 1.199 |  | 1 | | <0.001 | | 1.558 |  | 1 | 0.001 | 1.795 |  | 1 | 0.004 | 1.558 | |  | 1 | <0.001 | | 2.243 | |  |
|  | 2-3 | <0.001 | 2.14 |  | 2-3 | 0.015 | 1.429 |  | 2-3 | | <0.001 | | 1.966 |  | 2-3 | 0.005 | 1.656 |  | 2-3 | <0.001 | 2.400 | |  | 2-3 | <0.001 | | 3.142 | |  |
|  | ≥4 | <0.001 | 3.08 |  | ≥4 | <0.001 | 2.629 |  | ≥4 | | <0.001 | | 3.298 |  | ≥4 | <0.001 | 3.854 |  | ≥4 | <0.001 | 4.800 | |  | ≥4 | <0.001 | | 8.359 | |  |
|  | Constant | <0.001 | 0.18 |  | Constant | <0.001 | 0.265 |  | Constant | | <0.001 | | 0.364 |  | Constant | <0.001 | 0.167 |  | Constant | <0.001 | 0.251 | |  | Constant | <0.001 | | 0.163 | |  |
| **2** | Age 18-29 | <0.001 |  | **2** | ACEs 0 | <0.001 |  | **2** | Age 18-29 | | <0.001 | |  | **2** | *Male | 0.042 | 0.773 | **2** | ACEs 0 | <0.001 |  | | **2** | ACEs 0 | <0.001 | |  | |  |
|  | 30-39 | 0.304 | 0.84 |  | 1 | 0.260 | 1.177 |  | 30-39 | | 0.041 | | 0.733 |  | ACEs 0 | <0.001 |  |  | 1 | 0.005 | 1.558 | |  | 1 | <0.001 | | 2.139 | |  |
|  | 40-49 | <0.001 | 0.41 |  | 2-3 | 0.057 | 1.328 |  | 40-49 | | 0.017 | | 0.699 |  | 1 | 0.001 | 1.784 |  | 2-3 | <0.001 | 2.105 | |  | 2-3 | <0.001 | | 2.731 | |  |
|  | 50-59 | <0.001 | 0.5 |  | ≥4 | <0.001 | 2.187 |  | 50-59 | | <0.001 | | 0.558 |  | 2-3 | 0.006 | 1.639 |  | ≥4 | <0.001 | 3.633 | |  | ≥4 | <0.001 | | 6.219 | |  |
|  | 60-69 | <0.001 | 0.36 |  | Treated fairly | 0.001 | 0.626 |  | 60-69 | | <0.001 | | 0.536 |  | ≥4 | <0.001 | 3.799 |  | Supportive friends | <0.001 | 0.361 | |  | Given opportunities | <0.001 | | 0.459 | |  |
|  | ACEs 0 | <0.001 |  |  | Constant | <0.001 | 0.302 |  | ACEs 0 | | <0.001 | |  |  | Constant | <0.001 | 0.164 |  | Constant | <0.001 | 0.339 | |  | Constant | <0.001 | | 0.188 | |  |
|  | 1 | 0.161 | 1.28 | **3** | *Male | 0.029 | 0.793 |  | 1 | | 0.002 | | 1.498 |  |  |  |  | **3** | ACEs 0 | <0.001 |  | | **3** | Deprivation (least) 1 | <0.001 | |  | |  |
|  | 2-3 | <0.001 | 1.95 |  | ACEs 0 | <0.001 |  |  | 2-3 | | <0.001 | | 1.857 |  |  |  |  |  | 1 | 0.01 | 1.499 | |  | 2 | 0.12 | | 1.481 | |  |
|  | ≥4 | <0.001 | 2.80 |  | 1 | 0.278 | 1.17 |  | ≥4 | | <0.001 | | 3.090 |  |  |  |  |  | 2-3 | <0.001 | 1.952 | |  | 3 | 0.089 | | 1.511 | |  |
|  | Constant | <0.001 | 0.17 |  | 2-3 | 0.068 | 1.312 |  | Constant | | <0.001 | | 0.360 |  |  |  |  |  | ≥4 | <0.001 | 2.953 | |  | 4 | <0.001 | | 2.639 | |  |
|  |  |  |  |  | ≥4 | <0.001 | 2.143 | **3** | Age 18-29 | | <0.001 | |  |  |  |  |  |  | Supportive friends | <0.001 | 0.439 | |  | (most) 5 | <0.001 | | 2.864 | |  |
|  |  |  |  |  | Treated fairly | 0.001 | 0.615 |  | 30-39 | | 0.025 | | 0.71 |  |  |  |  |  | Role model | <0.001 | 0.523 | |  | ACEs 0 | <0.001 | |  | |  |
|  |  |  |  |  | Constant | <0.001 | 0.298 |  | 40-49 | | 0.015 | | 0.695 |  |  |  |  |  | Constant | <0.001 | 0.385 | |  | 1 | <0.001 | | 2.101 | |  |
|  |  |  |  |  |  |  |  |  | 50-59 | | <0.001 | | 0.546 |  |  |  |  | **4** | Age 18-29 | 0.003 |  | |  | 2-3 | <0.001 | | 2.596 | |  |
|  |  |  |  |  |  |  |  |  | 60-69 | | <0.001 | | 0.527 |  |  |  |  |  | 30-39 | 0.075 | 0.735 | |  | ≥4 | <0.001 | | 5.803 | |  |
|  |  |  |  |  |  |  |  |  | *Male | | <0.001 | | 0.686 |  |  |  |  |  | 40-49 | 0.002 | 0.581 | |  | Given opportunities | <0.001 | | 0.467 | |  |
|  |  |  |  |  |  |  |  |  | ACEs 0 | | <0.001 | |  |  |  |  |  |  | 50-59 | <0.001 | 0.523 | |  | Constant | <0.001 | | 0.179 | |  |
|  |  |  |  |  |  |  |  |  | 1 | | 0.003 | | 1.483 |  |  |  |  |  | 60-69 | 0.015 | 0.657 | | **4** | Deprivation (least) 1 | <0.001 | |  | |  |
|  |  |  |  |  |  |  |  |  | 2-3 | | <0.001 | | 1.831 |  |  |  |  |  | ACEs 0 | <0.001 |  | |  | 2 | 0.139 | | 1.455 | |  |
|  |  |  |  |  |  |  |  |  | ≥4 | | <0.001 | | 3.029 |  |  |  |  |  | 1 | 0.022 | 1.438 | |  | 3 | 0.098 | | 1.493 | |  |
|  |  |  |  |  |  |  |  |  | Constant | | <0.001 | | 0.350 |  |  |  |  |  | 2-3 | <0.001 | 1.877 | |  | 4 | <0.001 | | 2.614 | |  |
|  |  |  |  |  |  |  |  |  |  | |  | |  |  |  |  |  |  | ≥4 | <0.001 | 2.843 | |  | (most) 5 | <0.001 | | 2.820 | |  |
|  |  |  |  |  |  |  |  |  |  | |  | |  |  |  |  |  |  | Supportive friends | <0.001 | 0.433 | |  | ACEs 0 | <0.001 | |  | |  |
|  |  |  |  |  |  |  |  |  |  | |  | |  |  |  |  |  |  | Role Model | <0.001 | 0.509 | |  | 1 | <0.001 | | 2.070 | |  |
|  |  |  |  |  |  |  |  |  |  | |  | |  |  |  |  |  |  | Constant | <0.001 | 0.386 | |  | 2-3 | <0.001 | | 2.446 | |  |
|  |  |  |  |  |  |  |  |  |  | |  | |  |  |  |  |  |  |  |  |  | |  | ≥4 | <0.001 | | 5.037 | |  |
|  |  |  |  |  |  |  |  |  |  | |  | |  |  |  |  |  |  |  |  |  | |  | Given opportunities | 0.001 | | 0.575 | |  |
|  |  |  |  |  |  |  |  |  |  | |  | |  |  |  |  |  |  |  |  |  | |  | Treated fairly | 0.003 | | 0.585 | |  |
|  |  |  |  |  |  |  |  |  |  | |  | |  |  |  |  |  |  |  |  |  | |  | Constant | <0.001 | | 0.199 | |  |
|  |  |  |  |  |  |  |  |  |  | |  | |  |  |  |  |  |  |  |  |  | | **5** | Age 18-29 | 0.045 | |  | |  |
|  |  |  |  |  |  |  |  |  |  | |  | |  |  |  |  |  |  |  |  |  | |  | 30-39 | 0.711 | | 1.079 | |  |
|  |  |  |  |  |  |  |  |  |  | |  | |  |  |  |  |  |  |  |  |  | |  | 40-49 | 0.927 | | 0.981 | |  |
|  |  |  |  |  |  |  |  |  |  | |  | |  |  |  |  |  |  |  |  |  | |  | 50-59 | 0.403 | | 1.185 | |  |
|  |  |  |  |  |  |  |  |  |  | |  | |  |  |  |  |  |  |  |  |  | |  | 60-69 | 0.029 | | 0.598 | |  |
|  |  |  |  |  |  |  |  |  |  | |  | |  |  |  |  |  |  |  |  |  | |  | Deprivation (least) 1 | <0.001 | |  | |  |
|  |  |  |  |  |  |  |  |  |  | |  | |  |  |  |  |  |  |  |  |  | |  | 2 | 0.153 | | 1.439 | |  |
|  |  |  |  |  |  |  |  |  |  | |  | |  |  |  |  |  |  |  |  |  | |  | 3 | 0.101 | | 1.491 | |  |
|  |  |  |  |  |  |  |  |  |  | |  | |  |  |  |  |  |  |  |  |  | |  | 4 | <0.001 | | 2.565 | |  |
|  |  |  |  |  |  |  |  |  |  | |  | |  |  |  |  |  |  |  |  |  | |  | (most) 5 | <0.001 | | 2.726 | |  |
|  |  |  |  |  |  |  |  |  |  | |  | |  |  |  |  |  |  |  |  |  | |  | ACEs 0 | <0.001 | |  | |  |
|  |  |  |  |  |  |  |  |  |  | |  | |  |  |  |  |  |  |  |  |  | |  | 1 | <0.001 | | 2.056 | |  |
|  |  |  |  |  |  |  |  |  |  | |  | |  |  |  |  |  |  |  |  |  | |  | 2-3 | <0.001 | | 2.325 | |  |
|  |  |  |  |  |  |  |  |  |  | |  | |  |  |  |  |  |  |  |  |  | |  | ≥4 | <0.001 | | 4.731 | |  |
|  |  |  |  |  |  |  |  |  |  | |  | |  |  |  |  |  |  |  |  |  | |  | Given opportunities | <0.001 | | 0.564 | |  |
|  |  |  |  |  |  |  |  |  |  | |  | |  |  |  |  |  |  |  |  |  | |  | Treated fairly | 0.003 | | 0.59 | |  |
|  |  |  |  |  |  |  |  |  |  | |  | |  |  |  |  |  |  |  |  |  | |  | Constant | <0.001 | | 0.196 | |  |

Final models are displayed in Table 4. Models were developed in SPSS using a conditional logistic regression model. AOR = Adjusted Odds Ratio; ACEs = Adverse childhood experience (ACE) count.

**Table S3. Prevalence of ACEs reported by age category of respondent**

|  |  | **Physical**  **abuse** | **Verbal**  **abuse** | **Sexual**  **abuse** | **Physical**  **neglect** | **Emotional**  **neglect** | **Parental**  **separation** | **Domestic**  **violence** | **Mental**  **illness** | **Alcohol**  **abuse** | **Drug**  **abuse** | **Incarce-ration** | **ACE count** | | | |
| --- | --- | --- | --- | --- | --- | --- | --- | --- | --- | --- | --- | --- | --- | --- | --- | --- |
|  |  |  |  |  |  |  |  |  |  |  |  |  | **0** | **1** | **2-3** | **≥4** |
| **Age**  **(years)** | 18-29 | 14.2 | 22.9 | 5.9 | 5.0 | 6.2 | 41.9 | 12.8 | 25.2 | 17.6 | 8.7 | 5.5 | 41.4 | 21.7 | 19.7 | 17.2 |
|  | 30-39 | 14.4 | 19.9 | 6.0 | 4.0 | 7.1 | 31.4 | 15.9 | 19.0 | 12.4 | 8.2 | 4.6 | 47.8 | 19.0 | 18.6 | 14.6 |
|  | 40-49 | 20.4 | 24.4 | 9.3 | 5.7 | 9.9 | 23.4 | 21.0 | 16.4 | 15.4 | 6.9 | 4.4 | 49.1 | 18.0 | 16.8 | 16.2 |
|  | 50-59 | 17.8 | 20.6 | 8.3 | 3.8 | 7.5 | 17.1 | 20.8 | 15.0 | 12.3 | 2.8 | 3.8 | 53.9 | 16.2 | 16.2 | 13.7 |
|  | 60-69 | 11.4 | 11.7 | 7.6 | 2.5 | 6.7 | 9.8 | 12.3 | 11.4 | 6.9 | 1.4 | 2.0 | 62.2 | 19.9 | 11.2 | 6.7 |
|  | *X^2^*_trend_ | 0.530 | 16.698 | 2.006 | 3.909 | 0.077 | 165.671 | 0.106 | 34.087 | 21.684 | 39.618 | 8.585 | 52.537 | | | |
|  | P | 0.467 | <0.001 | 0.211 | 0.048 | 0.782 | <0.001 | 0.744 | <0.001 | <0.001 | <0.001 | 0.003 | <0.001 | | | |

See Table S1 for full description of each ACE.
